# Serious Games for Nutritional Education: Online Survey on Preferences, Motives, and Behaviors Among Young Adults at University

**DOI:** 10.2196/16216

**Published:** 2020-06-03

**Authors:** Sophie Laura Holzmann, Hanna Schäfer, David Alexander Plecher, Lynne Stecher, Gudrun Johanna Klinker, Georg Groh, Hans Hauner, Christina Holzapfel

**Affiliations:** 1 Institute for Nutritional Medicine, Else Kröner-Fresenius-Center for Nutritional Medicine School of Medicine Technical University of Munich Munich Germany; 2 Research Group Social Computing Department of Informatics Technical University of Munich Garching Germany; 3 Chair for Computer Aided Medical Procedures & Augmented Reality Department of Informatics Technical University of Munich Garching Germany

**Keywords:** nutrition, information sources, serious games, digital gameplay, preferences, motives, behavior, university students, survey

## Abstract

**Background:**

Data on nutritional information and digital gameplay are limited among young adults in Germany.

**Objective:**

This survey aimed to gather data on nutritional information sources and digital games for nutritional education (preferences, motives, and behaviors) among young adults at both Munich universities in Germany.

**Methods:**

An online survey was developed by an multidisciplinary research group using EvaSys, an in-house survey software. The questionnaire (47 items) covered questions about baseline characteristics (eg, housing situation and weight), nutrition (eg, nutritional information sources), and digital (nutritional) gameplay (eg, preferences, motives, and behaviors). A feedback field was also provided. This publication is based on a selection of 20 questions (7 baseline characteristics, 2 nutrition, 11 gameplay). Young adults, primarily Munich university students aged from 18 to 24 years, were invited to participate by digital and nondigital communication channels between 2016 and 2017. Statistical analyses were performed using Excel 2013 (Microsoft Corp) and R version 3.1.3 (R Foundation for Statistical Computing).

**Results:**

In total, 468 young adults (342/468, 73.1% women; 379/468, 81.0% university students) participated. Most of the participants (269/468, 57.5%) were aged 18 to 24 years with a BMI in the normal weight range (346/447, 77.4%). Mean body weight was 65.5 [SD 14.0] kg. Most participants reported getting nutritional information from the internet (372/467, 79.7%) and printed media (298/467, 63.8%), less than 1.0% (2/467, 0.4%) named digital games. Apps (100/461, 21.7%) and university/workplace (146/461, 31.7%) were the most desired sources for additional information about nutrition, while 10.0% (46/461, 10.0%) of participants stated wanting digital games. Almost two-thirds (293/468, 62.6%) of participants played digital games, while one-fifth (97/456, 21.3%) played digital games daily using smartphones or tablets. Finally, most respondents (343/468, 73.3%), mainly women, expressed interest in obtaining nutritional information during digital gameplay. However, significant gender differences were shown for nutritional acquisition behaviors and digital gameplay preferences, motives, and behaviors.

**Conclusions:**

Our survey population reported playing digital games (especially men) and wanting nutritional information during digital gameplay (especially women). Furthermore, university or workplace are named as preferred settings for nutritional information. Therefore, a digital game app might have the potential to be a tool for nutritional education among young adults within the university or workplace environment.

## Introduction

### Background

The prevalence of overweight and obesity reached global pandemic and represents a major public health concern [1]. In Germany, 7.2% of young adults (aged 18 to 24 years) are affected by obesity [2]. Moreover, it is reported that young adults of Western nations are more vulnerable to weight gain than any other age group [3]. Obesogenic environments with increases in energy-dense foods and sedentary behaviors are the main causes for the development of overweight and obesity [1,3]. According to the German National Consumption Study II, adolescents (aged 15 to 18 years) and young adults (aged 19 to 24 years) show the highest daily intake of free sugar [4]. This also applies at the global level and for other nutrients or food groups (eg, low intake of fruits and vegetables) [3,5-7].

To address overweight and obesity among young adults, nutritional education programs can be used within different settings (eg, workplace, home, community, health care, and educational facilities) at both the individual and population level [1,8,9]. These programs are applied to change knowledge, attitudes, and beliefs about healthy dietary behaviors and also affect the dietary behaviors themselves [10-12]. For instance, serious games present a novel digital approach to nutritional education by conveying nutritional information in an entertaining format [13,14]. In recent years, the market for games and number of gaming individuals has increased. In 2017, 34.1 million people in Germany played computer and video games [15]. The literature indicates that serious gameplay might be an appealing and effective tool for nutritional education and dietary behavior change [14,16]. A few studies investigated the effects of serious games on nutritional education among (young) adults [12]. According to a narrative review, nutritional knowledge can be increased in young adults (aged 18 to 35 years) through game-based nutritional interventions [12]. A randomized controlled study among 40 persons (80% women; mean age 20 years) showed that playing a role-play computer game for 3 weeks improved knowledge about nutrition and weight management for the short term [17]. According to a 3-month study among 47 women with an average age of 30 years, playing a serious computer game resulted in significant improvements of body mass index and nutritional knowledge from baseline to the end of the study [18].

### Aim

The aim of this online survey was to collect data on young adults’ nutritional information sources and preferences, motives, and behaviors regarding a digital game for nutritional education purposes.

## Methods

### Recruitment

The Ethical Committee of the School of Medicine at the Technical University of Munich approved this open online survey (ethical vote: 164/16S), which was performed from December 2016 to July 2017 in Munich, the third largest city in Germany, with approximately 1.5 million inhabitants. The survey invitation included a password and link guiding participants to the online survey. The recruitment of young adults was conducted mainly through digital communication channels (eg, newsletters, social media, and email) at both Munich universities (Technical University of Munich and Ludwig-Maximilians University) and primarily included students, graduates, and employees. Furthermore, recruitment was extended by social media (eg, Facebook), printed flyers (eg, at canteens), and other student-affiliated distribution channels (eg, Technical University of Munich General Student Committee).

Since the exact number of invitations is unknown, it was not possible to calculate a response rate. The following inclusion criteria were applied: understanding of the German language, willingness to participate, and internet access. All participants gave informed consent to participate.

The first page of the survey provided information about the research team, aim of the survey, target group (young adults aged from 18 to 24 years), and instruction (eg, completion time of 5 to 10 minutes). Finally, information about data privacy and protection (eg, voluntary participation confidential, no personal data) was provided. Participants had to confirm the data privacy statement before starting the survey. Each question had to be answered to continue. No incentives were offered to the participants.

### Questionnaire: Development and Design

The 47-item questionnaire was developed by an multidisciplinary team of nutritionists, economists, sociologists, and computer scientists using EvaSys V7.0 (2101) survey software. Questions (closed, semi-open, open; single or multiple choice) referred to nutrition (16 questions), digital games (22 questions), personal characteristics (8 questions), and feedback (1 field). Wherever possible, a “don’t know,” “no answer applies,” or “other” option was provided. This questionnaire represents a revised version of a questionnaire developed by the same multidisciplinary research team and previously used in a survey among children and adolescents [19]. Age-specific modifications were made to ensure age adequacy. Similar to our previous survey [19], this work is based on a selection of 20 (7 baseline characteristics, 2 nutrition, 11 gameplay) of the 47 questions. Questions not presented were mainly focused on technical issues for design and development of a game.

### Statistical Analyses

Only completed questionnaires were analyzed. Integrity and plausibility checks were performed. Questionnaires with missing answers for 8 or more questions or invalid answers were excluded. Descriptive data analyses (frequencies, percentages, standard deviations, and means) were performed using Excel 2013 (Microsoft Corp). Gender differences in responses were assessed using Pearson chi-square tests for categorical variables. These analyses were performed using R version 3.1.3 (R Foundation for Statistical Computing). *P* values of <.05 were considered statistically significant. The analyses are based on 20 out of 47 questions.

## Results

### Participant Characteristics

Table 1 presents the sociodemographic and anthropometric characteristics of the participants. In total, 468 young adults (342/468, 73.1% women), primarily university students (379/468, 81.0%), participated. Nonstudent participants were mainly those previously affiliated with a university (eg, graduates) or academic staff (eg, research assistants). Most participants were aged between 18 and 24 years (269/468, 57.5%). Body weight ranged from 35.9 kg to 190.0 kg (mean 65.5 [SD 14.0] kg) and height varied between 1.50 m and 2.02 m (mean 1.68 [SD 0.09] m). According to the body mass index (mean 22.3 [SD 3.6] kg/m^2^), participants were mostly normal weight (346/447, 77.4%). Most participants either lived with their family (193/468, 41.2%) or in a flat/house share (166/468, 35.5%).

**Table 1:** Characteristics of survey participants.

Characteristics			Value, n (%)
**Sociodemography (n=468)**			
	**Age in years**		
		18-19	24 (5.1)
		20-21	84 (18.0)
		22-24	161 (34.4)
		>24	199 (42.5)
	**Sex**		
		Female	342 (73.1)
		Male	126 (26.9)
	**Home environment**		
		Family	193 (41.2)
		Flat/house share	166 (35.5)
		Alone	95 (20.3)
		Other	14 (3.0)
**Anthropometry (n=447)**			
	**Body mass index**		
		Underweight (<18.5 kg/m^2^)	34 (7.6)
		Normal weight (18.5-24.9 kg/m^2^)	346 (77.4)
		Overweight (25.0-29.9 kg/m^2^)	54 (12.1)
		Obesity (≥30.0 kg/m^2^)	13 (2.9)

### Sources of Nutritional Information

Table 2 shows digital and nondigital sources of nutritional information that participants currently use or desire to additionally use. Most participants (372/467, 79.7%) responded that they use the internet for obtaining digital nutritional information (nonsignificant gender differences) with one-third (160/467, 34.4%) reported using social networks (significantly more women than men; *P*=.001).

Regarding nondigital sources, the majority (298/467, 63.8%) of respondents stated that they were informed about nutrition via books and newspapers (significantly more women than men; *P*<.001). The second most currently used nondigital information sources were partner and family (218/467, 46.7%) and friends (201/467, 43.0%; nonsignificant gender differences for both items), followed by university and workplace (164/467, 35.1%; significantly more women than men; *P*=.001).

More than a fifth (100/461, 21.7%) of participants would like to receive additional digital nutritional information via apps (nonsignificant gender differences). Television (67/461, 14.5%), internet (65/461, 14.1%), and social networks (71/461, 15.4%) were each requested as nutritional information sources by almost 15% of participants. Significant gender differences occurred for social networks only (significantly more women than men; *P*=.04). The main desired nondigital source for additional information about nutrition was university and workplace (146/461, 31.7%) followed by books and newspapers (52/461, 11.3%). No statistically significant differences were found between women and men.

Specifically regarding digital games, only two participants (2/467, 0.4%) responded that they are currently using digital games for nutritional information (nonsignificant gender differences), but 10.0% (46/461) would like to use digital games as an additional nutritional information source (significantly more women than men; *P*=.003).

**Table 2:** Digital and nondigital sources of nutritional information (multiple responses allowed).

Source and characteristic			All, n (%)	Female, n (%)	Male, n (%)	*P* value
**Currently used^a^ (n=467)**						
	**Digital**					
		Television	98 (21.0)	73 (21.3)	25 (20.0)	.75
		Internet	372 (79.7)	271 (79.2)	101 (80.8)	.71
		Social networks	160 (34.4)	132 (38.6)	28 (22.4)	.001
		Apps	66 (14.1)	61 (17.8)	5 (4.0)	<.001
		Digital games	2 (0.4)	2 (0.6)	0 (0)	.39
	**Nondigital**					
		University and workplace	164 (35.1)	135 (39.5)	29 (23.2)	.001
		Partner and family	218 (46.7)	153 (44.7)	65 (52.0)	.16
		Friends	201 (43.0)	153 (44.7)	48 (38.4)	.22
		Books and newspapers	298 (63.8)	237 (69.3)	61 (48.8)	<.001
	**Other**					
		Unknown	109 (23.3)	81 (23.7)	28 (22.4)	.77
		No answer applies	7 (1.5)	3 (0.9)	4 (3.2)	.07
**Additionally desired^b^ (n=461)**						
	**Digital**					
		Television	67 (14.5)	54 (16.1)	13 (10.4)	.13
		Internet	65 (14.1)	44 (13.1)	21 (16.8)	.31
		Social networks	71 (15.4)	59 (17.6)	12 (9.6)	.04
		Apps	100 (21.7)	78 (23.2)	22 (17.6)	.19
		Digital games	46 (10.0)	42 (12.5)	4 (3.2)	.003
	**Nondigital**					
		University and workplace	146 (31.7)	108 (32.1)	38 (30.4)	.72
		Partner and family	30 (6.5)	24 (7.1)	6 (4.8)	.37
		Friends	29 (6.3)	23 (6.8)	6 (4.8)	.42
		Books and newspapers	52 (11.3)	42 (12.5)	10 (8.0)	.18
	**Other**					
		Unknown	40 (8.7)	35 (10.4)	5 (4.0)	.03
		No answer applies	173 (37.5)	111 (33.0)	62 (49.6)	.001

^a^How are you currently informed about nutrition?

^b^How would you like to be additionally informed about nutrition?

### Digital Gameplay: Preferences, Motives, and Behaviors

Table 3 shows that more than one-half of the participants (293/468, 62.6%) reported playing digital games. Men were significantly more likely to play digital games (100/126, 79.4%) compared with women (193/342, 56.4%; *P*<.001). The most frequent answer regarding the preferred team composition within a digital game was friends (274/464, 59.1%). A similar proportion of participants responded with partner and family (193/464, 41.6%) and individuals with the same eating behavior (188/464, 40.5%). There were no statistically significant differences between women and men.

The questionnaire also asked for the most preferred game character in digital games. Nearly half of participants (226/468, 48.3%) preferred a human being while almost a fifth (89/468, 19.0%) preferred a fantasy character (nonsignificant gender differences), followed by a cute animal (51/468, 10.9%), which was significantly more desired by women than by men (*P*=.01).

In addition to these preferences, Table 3 presents the frequency of digital gameplay by device. The most frequent answer of participants who reported playing digital games on smartphones or tablets was daily (97/456, 21.3%), followed by weekly (66/456, 14.5%) and monthly (65/456, 14.3%), with no statistically significant differences between women and men. Half of the participants (222/453, 49.0%), who use digital games, reported short gameplay (≤30 minutes).

One-fifth (99/463, 21.4%) of participants reported that they play digital games for more than 60 minutes continuously on PCs or consoles, and one-eighth (57/463, 12.3%) reported doing so for exactly 60 minutes (significantly more men than women: *P*<.001 vs *P*=.003).

More than 40% of the participants reported mainly playing digital games if they felt like gaming (199/442, 45.0%; significantly more men than women; *P*<.001) or were bored (190/442, 43.0%; nonsignificant gender differences*; P*=.13). In total, 10.4% (46/442) of participants reported playing digital games when they are happy (significantly more men than women; *P*<.001). Moreover, one-third of participants (153/442, 34.6%) reported playing digital games often alone at home (significantly more men than women; *P*=.003), and one-fifth (101/442, 22.9%) stated that they played digital games mainly on the go (significantly more women than men; *P*=.03). Significantly more men than women reported playing digital games with friends (*P*<.001).

Participants were asked whether they would like to receive nutritional information by playing digital games. Nearly two-thirds (293/468, 62.6%) of participants expressed an interest in receiving nutritional information during gameplay, with women reporting this significantly more often than men (*P*<.001). Moreover, almost three-quarters indicated they preferred being educated via answering quiz questions (323/463, 69.8%) or completing tasks (333/463, 71.9%) during digital gameplay, with significantly more women than men reporting both of these (quiz *P*<.001 vs task *P*=.001). In contrast, significantly more men than women stated that they don’t want to learn anything during digital gameplay (*P*<.001).

**Table 3:** Digital gameplay: preferences, motives, and behaviors.

Digital gameplay and characteristics			All, n (%)		Female, n (%)		Male, n (%)		*P* value	
**Preferences**										
	**Team composition^a,b^ (n=464)**									
		Partner and family		193 (41.6)		144 (42.5)		49 (39.2)		.53
		Friends		274 (59.1)		199 (58.7)		75 (60.0)		.80
		Same hobbies		132 (28.4)		97 (28.6)		35 (28.0)		.90
		Same eating behavior		188 (40.5)		144 (42.5)		44 (35.2)		.16
		No similarities		109 (23.5)		79 (23.3)		30 (24.0)		.88
		Other		26 (5.6)		20 (5.9)		6 (4.8)		.65
		No team		37 (8.0)		25 (7.4)		12 (9.6)		.43
	**Teammates^c,d^ (n=445)**									
		≤5		116 (26.1)		67 (20.7)		49 (40.2)		<.001
		6-10		11 (2.5)		5 (1.5)		6 (4.9)		.04
		>10		8 (1.8)		3 (0.9)		5 (4.1)		.03
		Alone		130 (29.2)		98 (30.3)		32 (26.2)		.40
		I do not play		152 (34.2)		129 (39.9)		23 (18.9)		<.001
		I do not know		28 (6.3)		21 (6.5)		7 (5.7)		.77
	**Game character^c,e^ (n=468)**									
		Cute animal		51 (10.9)		45 (13.2)		6 (4.8)		.01
		Impressive animal		23 (4.9)		16 (4.7)		7 (5.6)		.70
		Fantasy animal		20 (4.3)		15 (4.4)		5 (4.0)		.84
		Fantasy character		89 (19.0)		62 (18.1)		27 (21.4)		.42
		Human being		226 (48.3)		163 (47.7)		63 (50.0)		.65
		Other		26 (5.6)		18 (5.3)		8 (6.3)		.65
		No answer applies		33 (7.1)		23 (6.7)		10 (7.9)		.65
**Motives^a,f^ (n=442)**										
	**Emotion**									
		Pleasure		199 (45.0)		123 (38.3)		76 (62.8)		<.001
		Happiness		46 (10.4)		20 (6.2)		26 (21.5)		<.001
		Sadness		20 (4.5)		13 (4.0)		7 (5.8)		.43
		Boredom		190 (43.0)		131 (40.8)		59 (48.8)		.13
	**Setting**									
		Friends		36 (8.1)		16 (5.0)		20 (16.5)		<.001
		On the go		101 (22.9)		82 (25.5)		19 (15.7)		.03
		University and workplace		44 (10.0)		32 (10.0)		12 (9.9)		.99
		Alone at home		153 (34.6)		98 (30.5)		55 (45.5)		.003
		Other situations		27 (6.1)		18 (5.6)		9 (7.4)		.47
	**Other**									
		I do not play		154 (34.8)		131 (40.8)		23 (19.0)		<.001
		I do not know		0 (0)		0 (0)		0 (0)		—
**Behaviors**										
	**Digital gameplay^c,g^ (n=468)**									
		Yes		293 (62.6)		193 (56.4)		100 (79.4)		<.001
		No		175 (37.4)		149 (43.6)		26 (20.6)		—
	**Duration of digital gameplay (smartphone/tablet)^c,h^ (n=453)**									
		≤30 minutes		222 (49.0)		160 (48.6)		62 (50.0)		.80
		60 minutes		5 (1.1)		5 (1.5)		0 (0.0)		.17
		>60 minutes		4 (0.9)		2 (0.6)		2 (1.6)		.31
		I do not play		212 (46.8)		155 (47.1)		57 (46.0)		.83
		I do not know		10 (2.2)		7 (2.1)		3 (2.4)		.85
	**Duration of digital gameplay (PC/console)^c,i^ (n=463)**									
		≤30 minutes		50 (10.8)		36 (10.7)		14 (11.1)		.90
		60 minutes		57 (12.3)		32 (9.5)		25 (19.8)		.003
		>60 minutes		99 (21.4)		45 (13.4)		54 (42.9)		<.001
		I do not play		248 (53.6)		218 (64.7)		30 (23.8)		<.001
		I do not know		9 (1.9)		6 (1.8)		3 (2.4)		.68
	**Frequency of digital gameplay (smartphone/tablet)^c,j^ (n=456)**									
		Daily		97 (21.3)		72 (21.6)		25 (20.3)		.76
		Weekly		66 (14.5)		47 (14.1)		19 (15.4)		.72
		Monthly		65 (14.3)		47 (14.1)		18 (14.6)		.89
		I do not play		220 (48.2)		163 (48.9)		57 (46.3)		.62
		I do not know		8 (1.8)		4 (1.2)		4 (3.3)		.14
	**Frequency of digital gameplay (PC/console)^c,k^ (n=466)**									
		Daily		39 (8.4)		17 (5.0)		22 (17.7)		<.001
		Weekly		57 (12.2)		25 (7.3)		32 (25.8)		<.001
		Monthly		97 (20.8)		64 (18.7)		33 (26.6)		.06
		I do not play		257 (55.2)		225 (65.8)		32 (25.8)		<.001
		I do not know		16 (3.4)		11 (3.2)		5 (4.0)		.67
	**Nutritional education by digital games^c,l^ (n=468)**									
		Yes		343 (73.3)		273 (79.8)		70 (55.6)		<.001
		No		125 (26.7)		69 (20.2)		56 (44.4)		—
	**Ways of nutritional education by digital games^a,m^ (n=463)**									
		Quiz		323 (69.8)		253 (74.9)		70 (56.0)		<.001
		Tasks		333 (71.9)		257 (76.0)		76 (60.8)		.001
		Movies		150 (32.4)		114 (33.7)		36 (28.8)		.32
		Mates		98 (21.2)		71 (21.0)		27 (21.6)		.89
		Other		56 (12.1)		38 (11.2)		18 (14.4)		.36
		No learning		31 (6.7)		12 (3.6)		19 (15.2)		<.001
		No answer applies		15 (3.2)		12 (3.6)		3 (2.4)		.54

^a^Multiple responses allowed.

^b^In a digital game about nutrition, goals are to be achieved together in a team. Who should the players on this team be?

^c^Single response allowed.

^d^How many players do you like to play digital games with?

^e^Which character would you most like to be in a digital game?

^f^When do you often play digital games?

^g^Do you play digital games (smartphone, PC, console, apps)?

^h^How long do you play digital games on your smartphone/tablet/apps without any interruption?

^i^How long do you play digital games on your PC/console without any interruption?

^j^How often do you play digital games on your smartphone/tablet/apps? "App” is not shown in table.

^k^How often do you play digital games on your PC/console?

^l^Would you like to receive nutrition information in a digital game?

^m^How would you like to learn about nutrition in a digital game?

## Discussion

### Principal Findings

This survey reports findings on nutritional information strategies and motives, preferences, and behaviors for digital gameplay among more than 450 young adults, mainly university students. In total, 62.6% (293/468) of the participants reported playing digital games. Our findings are in line with data from the United States. According to data from the Pew Research Center, 60% of young American adults aged between 18 and 29 years play video games often or sometimes (computer, television, game console, portable device), with young men more likely to play video games than young women [20,21]. In contrast, data from an online survey among 900 Finish and Belgian university students and employees (mean age 26.8 years) showed that only one-third of participants play mobile games [22].

Regarding device preferences, more participants reported using smartphones or tablets than PCs or consoles on a daily and weekly basis. These findings are different than results from a survey among 292 participants at a US gaming convention (68.6% male; mean age 34.2 [SD 10.6] years) showing that the computer was the preferred platform for gameplay [23]. According to the literature, smartphones, PCs, and consoles are the most preferred gaming platforms in Germany [24].

Most of the survey sample who play digital games reported doing so for more than one hour continuously on PCs or consoles. These findings are similar to data from Arnaez et al [23] demonstrating that American adults spent on average at least 2 hours per day on computers or consoles for gameplay. Lopez-Fernandez et al [22] revealed that the average time of smartphone gameplay was almost 2 hours per day, which is longer than our survey participants indicated.

### Nutritional Information: Interest and Acquisition

This survey revealed that the most used nutritional information sources currently were internet (79.7%) and books and newspapers (63.8%), followed by partner and family (46.7%), friends (43.0%), university and workplace (35.1%), and social networks (34.4%). Findings are in line with results from a cross-sectional study about nutritional information sources among 192 Ghanaian young adults (51.0% women; 66% students) aged between 18 and 25 years [25]. The study showed that the most consistently used nutritional sources were online resources (92.7%), followed by traditional media (58.3%), peers and friends (29.7%), family members (29.7%), and health care professionals (4.7%) [25]. According to a survey by Percheski and Hargittai [26], social networks (89.5%) were the most used source of health information among 1060 US university students, followed by online (78.4%), medical professionals (75.5%), and traditional media (74.6%). With respect to the interpretation and comparison of our survey with other data, it must be acknowledged that the classification of nutritional information sources into digital and nondigital varies between studies. In addition, there might be some overlaps regarding the single items—for instance, the social networks resource might include digital resources (eg, internet, apps) or nondigital ones (eg, partner and family, friends). Furthermore, the subanalysis showed that there are gender-specific differences in the acquisition of nutritional information among our survey population. The literature on health information–seeking behaviors among university students in the United States indicates that women are more likely than men to use online resources or seek health information in general [26-28], which also applies to our survey results. In general, this survey as well as the literature indicate that both digital and nondigital tools and settings are used and desired for nutrition information. Considering this aspect, digital and nondigital nutrition information sources complement one another and should be offered in parallel.

It was shown that nearly three-quarters (73.3%) of participants indicated they were interested in receiving nutritional information through digital games (Table 3). This is in line with a focus group study showing that young adults (mean age 23.1 [SD 2.7] years) perceive mobile games as a reasonable nutritional education approach [6]. According to a previous survey, this interest is lower in children and adolescents [19]. In total, 0.4% of participants reported currently using digital games for obtaining nutritional information and 10.0% reported desiring them additionally. When asked whether they would like to receive nutritional information during digital gameplay, more than 70% of participants, especially women, responded yes. This question was asked at the beginning of the gameplay section, where we added a scenario to allow participants to better immerse themselves in the questions. However, the scenario might have biased participant response behavior. Finally, we assume the responses are not in contrast to each other.

Until now, data regarding the sources of nutritional information in Germany has been limited. According to a survey on German dietary and shopping behaviors conducted on behalf of the Federal Ministry of Food and Agriculture in 2017, 69% of participants preferred receiving nutritional information personally at the point of sale, whereas 42% preferred an internet/online search [29]. Social media were used for information by 14% (31% aged from 18 to 29 years) of the survey population. [29]. These findings are in line with this survey revealing that digital nutritional information is collected primarily via the internet and social networks, while books and newspapers, partner and family, and friends were the most preferred nondigital sources.

The relevance of nondigital information channels was confirmed by the German federal report [29]. European data indicates a similar picture as friends and newspapers are the most frequently used nutritional information sources for adults [30]. Our findings are in line with data from Canada demonstrating that 94% of households count at least one person using the internet for health guidance [31]. According to a cross-sectional study with almost 200 young adults, online resources were the most popular nutritional information sources, while family members and friends/peers played a minor role [25]. Moreover, most participants indicated that the most reliable sources for nutritional information are health care professionals [25]. As the option nutritional expert was not provided within this survey, there are no comparable data available.

Most survey participants reported wanting digital nutritional information sources (television, internet, social networks, apps, and digital games) in addition to the sources currently used (Table 2). This finding might be of added value in terms of developing a digital nutritional game for adults, as online information resources are available and accessible for myriad demographics with a simple dissemination [25]. Therefore, it could be assumed that young adults may be a suitable target group for nutritional education through serious games.

Since consideration of the target groups’ needs, interests, perspectives, and preferences is crucial for the development of effective health games [16,32], data about learning preferences were collected in this survey as well. The majority of participants stated wanting to learn about nutrition in a digital game by playing a quiz or by solving tasks, which is consistent with data among young adults [6] and the younger population [19].

Our data showed that adults mainly prefer a human being or fantasy character in digital games. The preference for fantasy contexts or fantasy characters in serious games has already been shown for children [13,19] and students [33]. Besides the motivational nature of fantasy [13], fantasy-related contents used in video games improved the transfer of nutritional knowledge in children significantly more than no fantasy contexts [34]. Presented preferences should be considered for the development and design of a game for young adults [35]. A review on the usability of health-related games indicated that one of the main limitations of games for health is their lack in customization [36].

### Strengths and Limitations

This survey addresses a rather large sample of young adults, mainly university students, an underrepresented target group in gaming research and nutritional education interventions [37,38]. Our overall purpose was to assess young adult preferences, motives, and behaviors regarding a serious game for nutritional education. The questionnaire was developed by a multidisciplinary team of scientists to ensure that perspectives of relevant disciplines were considered. Since there was no validated questionnaire available for the purpose of our survey, we developed a questionnaire from scratch according to common recommendations. A previous version of the questionnaire was pretested internally by university students. However, there are some limitations that need to be mentioned. The questionnaire also covered technical game design aspects, and we refrained from presenting these data. Moreover, there might have been some overlaps regarding the response items within the questionnaire (eg, Table 2, questions 1 and 2), because participants might be not able to distinguish between, for instance, apps and digital games, since digital games can be apps and vice versa. This might be an explanation for the observation that only a few participants used (0.4%) or wanted (10.0%) a digital game for nutritional information, but almost three-quarters (73.3%) wanted nutritional information during the gameplay of a serious nutritional game. Another explanation for this result might be that questions were asked in different contexts. Moreover, data regarding digital media consumption (frequency and duration) might be limited, since we did not discriminate between digital gameplay on weekdays and weekends. The online survey might be prone to selection bias, as participation is limited to those with internet access [39,40]. Furthermore, the survey invitation was mainly distributed via university-associated channels in the large city of Munich (Bavaria) resulting in more than 80% university students. Because the survey was not restricted to university students, nonstudents also participated. The majority of nonstudent participants indicated they were an academic (eg, graduates) or academic staff (eg, research assistants). Moreover, the sample size is mainly female, with more than 70% women. Therefore, presented data (eg, body weight, body mass index) are not representative for young adults or university students in Germany. All data were obtained by self-report.

### Implications for Research and Practice

This survey revealed novel findings regarding digital gameplay in the context of nutritional education among a survey sample of more than 450 young adults, primarily female university students, in Germany. Since the game design process often lacks in the consideration of the target group’s preferences, referring to this survey data might be useful for the design of a target group–specific game for young adults, especially females. Further aspects such as behavioral change techniques (eg, goal setting, self-monitoring) should be incorporated into the development of educational games. Moreover, previous studies have found young adults prefer simple and quick interfaces, gaming reward strategies, competitive incentives (eg, leader boards), and notifications [6].

As digital gameplay can be affected by high attrition rates, the implementation of different game elements (eg, simulation, adventure, and quizzes) should be considered. Compared with quizzes and adventures, simulations are perceived as more beneficial for educational purposes by students aged 18 to 33 years [41]. Finally, the target group’s preferences need to be considered during all development and design stages of serious games [16].

Since the scientific evidence on the effects and effectiveness of serious games on nutritional outcomes (eg, knowledge, behavior) among young adults is limited, there is a need for addressing this topic within high-quality and long-term studies in the future [12,18]. Behavior changes are multifactorial processes. Therefore, it remains unclear whether an increase in nutritional knowledge can affect dietary behaviors at all. For instance, literature on students (mean age 21.8 [SD 1.9] years) showed that knowledge of healthy foods does not lead to the consumption of healthy snacks [42].

The design and development of serious games is a cost- and time-intensive process that requires the involvement of both experts and target groups [43,44]. Baranowski et al [43] reported that it takes three-and-a-half years to develop a health-related behavior change game. Serious games released by commercial vendors might be more attractive to players because of high-end graphical user interfaces, multiple levels, animation, and interactivity than their counterparts from research facilities. This may also have an impact on the adherence since fun affects the intrinsic motivation and intention to change behavior.

### Conclusions

This survey showed that mainly male university students play digital games, but they are less willing to get nutritional education via serious games compared with female students. Nondigital tools and settings like books, newspapers, university, and workplace are used and desired for nutritional education by young adults. A digital game might have potential as a tool for nutritional information among both male and female young adults and university students, for instance, in the workplace and university environment. Further research among representative survey samples is warranted to draw final and generalizable conclusions regarding the demand and acceptability of serious games in the context of nutritional information. Finally, further research should address gender-specific differences in nutritional information acquisition and gameplay preferences, motives, and behaviors.
